# Concordance analysis of DNA and RNA profiling: The MD Anderson IMPACT2 study in precision oncology

**DOI:** 10.1038/s41392-026-02580-0

**Published:** 2026-02-24

**Authors:** Stephanie T. Schmidt, Mehmet A. Baysal, Siqing Fu, David S. Hong, Sarina A. Piha-Paul, Aung Naing, Jordi Rodon Ahnert, Timothy A. Yap, Ecaterina Elena Dumbrava, Jennifer Beck, Funda Meric-Bernstam, Apostolia Maria Tsimberidou

**Affiliations:** 1 Department of Genomic Medicine, The University of Texas MD Anderson Cancer Center, Houston, TX 77030 USA; 2 Institute for Data Science in Oncology, The University of Texas MD Anderson Cancer Center, Houston, TX 77030 USA; 3 Department of Investigational Cancer Therapeutics, The University of Texas MD Anderson Cancer Center, Houston, TX 77030 USA

**Keywords:** Translational research, Cancer genomics

## Abstract

DNA profiling is an established method for cancer treatment selection, while RNA profiling remains investigational. We explored associations between DNA and RNA alterations and between the number of genes with altered expression and overall survival (OS) using patient data from IMPACT2 (NCT02152254), a randomized study evaluating molecular profiling for guiding cancer therapy across tumor types. Molecular profiling, including DNA next-generation sequencing, was performed on all 829 patients in the IMPACT2 study. RNA profiling was performed by Tempus for 253 of 829 patients. We evaluated the concordance between DNA and RNA profiling, analyzed OS in 217 treated patients with RNA profiling, and assessed PD-L1 status and number of genes with altered expression. Fifty patients exhibited 58 concordant events, i.e., genomic and expression alteration(s) in the same gene, including 38 copy number events, and 41 patients had statistically significant concordance. We identified 123 gene pairs with significant associations between genomic and expression alterations (*p* < 0.05), including *TP53* alterations with *VEGFA* overexpression. The median OS for patients with 0–2, 3–5, and ≥6 genes with altered expression was 9.8, 11.9, and 6.7 months, respectively (*p* = 0.03). These results underscore RNA profiling’s potential actionability, and altered expression in ≥6 genes was associated with shorter OS. Significant concordance of *TP53* alterations with *VEGFA* overexpression may partially explain tumor response to bevacizumab in *TP53*-mutant patients.

## Introduction

The integration of molecular diagnostics into oncology has revolutionized the clinical management of patients with advanced cancers. Over the past decade, DNA-based profiling of both tumor and blood samples has become a cornerstone of precision oncology, enabling the identification of targetable genomic alterations and informing the selection of therapies based on the genomic landscape of tumors. This paradigm has led to the US Food and Drug Administration (FDA) approval of 138 targeted agents (as of April 2025) for the treatment of patients with solid tumors based on specific somatic mutations, gene fusions, or copy number alterations detected through DNA sequencing. Examples include *EGFR* mutations for tyrosine kinase inhibitors in non-small cell lung cancer (NSCLC), *BRAF* V600E mutations for BRAF inhibitors in melanoma and colorectal cancer, and *ALK* or *ROS1* rearrangements guiding the use of ALK inhibitors.^1–3^

Despite this progress, the clinical utility of DNA profiling alone is limited. Many patients harbor molecular alterations, the functional status of which may have an impact on the patient’s clinical outcomes. Moreover, not all patients have actionable DNA mutations.^4^ These factors have sparked interest in RNA expression profiling, which can capture transcriptional activity and downstream pathway activation, providing a deeper understanding of tumor biology than DNA profiling alone.

While RNA profiling is currently considered exploratory in most clinical settings, its potential to enhance biomarker discovery, refine therapeutic selection, and be used as a prognostic tool presents an important opportunity to improve patient outcomes. Unlike static DNA alterations, RNA signatures can reflect dynamic tumor status, immune microenvironment interactions, and pathway-level alterations.^5^ However, no RNA-based profiling assays have received FDA approval for therapy selection, and the integration of RNA profiling into routine clinical decision-making remains limited. A key barrier to adoption of RNA profiling is the lack of prospective clinical data validating the prognostic or predictive value of RNA alterations, especially when evaluated alongside DNA findings.

Some investigators have reported concordance between DNA and RNA alterations, with some oncogenes (e.g., *ERBB2*, *CCND1*) showing consistent overexpression when amplified; however, others have demonstrated poor correlation between DNA and RNA expression owing to regulatory, epigenetic, or post-transcriptional factors.^6–8^ Furthermore, the prognostic implications of global RNA expression alterations—such as the number of dysregulated genes per patient, which we term tumor transcriptional burden (TTB)—have not been well established in the context of prospective clinical trials.

To address these challenges, we conducted a post hoc analysis of patients enrolled in IMPACT2 (NCT02152254), a randomized, prospective study evaluating molecular profiling to guide therapy in advanced cancer. We examined the concordance between DNA and RNA alterations and explored whether TTB was associated with overall survival (OS) in patients with advanced cancer across tumor types.

## Results

### Patient demographics and baseline characteristics

Overall, 829 patients were enrolled in the IMPACT2 study. In Part A, molecular profiling was performed by Foundation Medicine from May 2014 until April 2017. RNA profiling was not available during that time period. In Part B, molecular profiling was performed by Tempus from April 2019 until October 2023. RNA profiling was performed after DNA next-generation sequencing and PD-L1 profiling were completed, if adequate tumor tissue was available.

Baseline characteristics of the 253 patients with RNA profiling results are summarized in Table 1. The median age was 60 years (range, 20 to 84 years), and 52% were women. The median number of prior therapies was 4; 223 (88%) patients had an ECOG performance status of 1, and the median number of metastatic sites was 2. Additionally, 27 (11%) patients had low albumin levels (<lower limit of normal), and 127 (50%) patients had high lactate dehydrogenase levels (≥upper limit of normal). The most common tumor types were colorectal cancer (21%), head and neck carcinoma (12%), and sarcoma (12%). DNA and RNA profiling (*N* = 253), altered gene expression (*N* = 237), and concordant events (*N* = 50) by tumor type are shown in Supplementary Table 1. Tissue samples used in DNA and RNA profiling were derived predominantly from the liver (*N* = 84), soft tissue (*N* = 54), lymph nodes (*N* = 47), and lung (*N* = 31), with the remainder from various organs (Supplementary Table 3).

**Table 1:** Baseline characteristics of patient who underwent profiling performed by Tempus, including both DNA and RNA profiling, and DNA profiling only

	Patients with Tempus profiling (*N* = 438)		Patients with DNA and RNA profiling (*N* = 253)		Patients with only DNA profiling (*N* = 185)		Chi-squared test^a^
Characteristic	Patients	%	Patients	%	Patients	%	*p*-value
Age ≥60 years	206	47	126	50	80	43	0.207
Male/Female	212/226	48/52	122/131	48/52	90/95	49/51	1
No. of prior therapies >3	214	49	128	51	86	46	0.452
ECOG 0/1	57/381	13/87	30/223	12/88	27/158	15/85	0.486
No. of metastatic sites >2	135	31	86	34	49	26	0.115
Albumin <3.5 g/dL	53	12	27	11	26	14	0.356
LDH high^b^	198	45	127	54	71	38	0.605
Tumor types							N/A
Brain	8	2	2	1	6	3	
Breast	40	9	24	9	16	9	
Colorectal	91	21	53	21	38	21	
Endocrine	9	2	5	2	4	2	
Gastrointestinal (non-colorectal)	52	12	23	9	29	16	
Genitourinary (non-prostate)	2	0.4	0	0	2	1	
Gynecologic (non-ovarian)	17	4	8	3	9	5	
Head and neck	44	10	30	12	14	8	
Lung	20	5	13	5	7	4	
Melanoma	21	5	15	6	6	3	
Mesothelioma	6	1	4	2	2	1	
Ovarian	14	3	9	4	5	3	
Pancreas	38	9	17	7	21	11	
Prostate	27	6	18	7	9	5	
Sarcoma	41	9	30	12	11	6	
Skin (non-melanoma)	2	0.4	1	0.3	1	0.5	
Unknown primary	5	1	1	0.3	4	2	
Other: Rosai Dorfman disease	1	0.2	0	0	1	0.5	

Baseline patient characteristics for those with profiling performed by Tempus (*N* = 438), both DNA and RNA profiling available (*N* = 253), and only DNA profiling available (*N* = 185)

*N/A* non-applicable

^a^Chi-squared testing was performed to compare patients with both DNA and RNA profiling available to those with only DNA profiling available

^b^LDH measurements were available in 235 patients. The remaining 63 patients included 18 who had both DNA and RNA profiling, and 45 patients who had only DNA profiling

We compared the baseline characteristics of the 253 patients with DNA and RNA profiling with those of the remaining 185 patients with insufficient tumor for RNA profiling (Table 1). No differences in demographic and baseline characteristics were observed between the two groups. Taken together, the 253 patients who underwent RNA profiling were representative of the 438 total patients in Part B.

## Distribution of alterations from DNA and RNA profiling

**Fig. 1:**
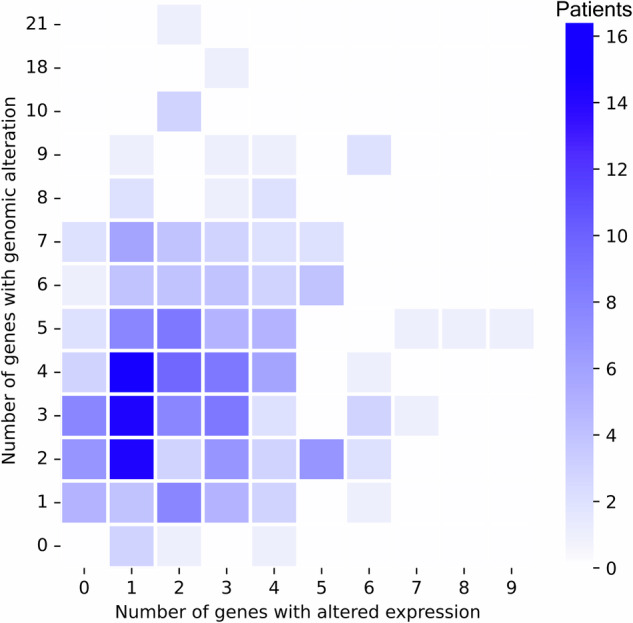
Distribution of number of genes with genomic alterations and number of genes with altered expression. Shading indicates the number of patients with the indicated count combination

The distribution of the number of alterations reported by DNA and RNA profiling for the 253 patients with RNA profiling is shown in Fig. 1. Genomic alterations were reported in 242 unique genes, and altered expression was reported in 52 unique genes. The median number of genes with genomic alterations in each patient was 4 (range, 0 to 21 genes), and the median number of genes with altered expression in each patient was 2 (range, 0 to 9 genes). The breakdown of events reported by DNA profiling was as follows: 42.9% were frameshift or stop-gain events, 30.4% were copy number events, 23.8% were single-nucleotide variants, and 2.9% were fusion or chromosomal rearrangement events. With respect to RNA profiling, 87.7% of events from RNA profiling reflected overexpression and 12.3% reflected underexpression (Supplementary Table 2).

### Concordance of alterations reported by DNA and RNA profiling

**Fig. 2:**
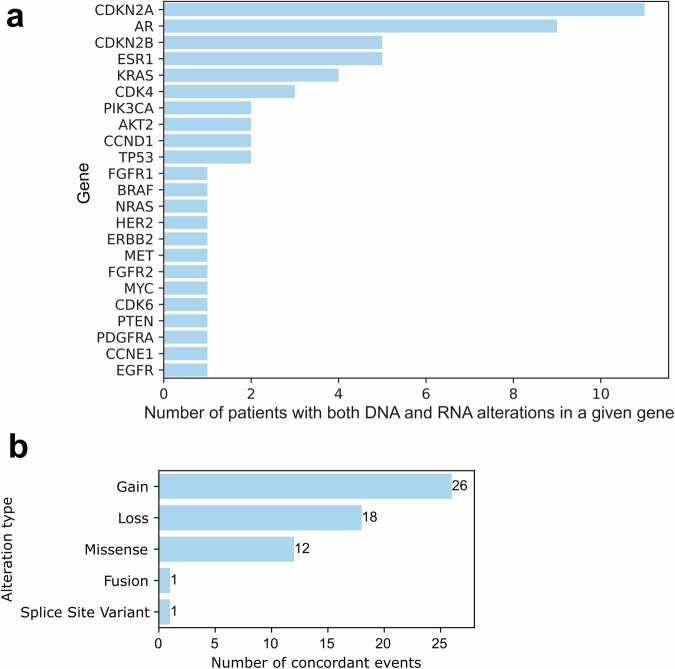
Concordant events observed by gene and alteration type. **a** Frequency of genes exhibiting concordance, i.e., alteration in the same gene reported by both DNA and RNA profiling. **b** Distribution of alteration types in concordant events

Altered genes identified by both DNA and RNA profiling in the same patient are shown in Fig. 2. Of the 253 patients with DNA and RNA profiling, 50 patients displayed concordance, i.e., genomic and expression alterations in the same gene. There were 58 concordant events among the 50 patients, with 44 patients exhibiting concordance in 1 gene, 4 patients exhibiting concordance in 2 genes, and 2 patients exhibiting concordance in 3 genes. The 58 concordant events involved 23 genes, and genes concordant in more than 1 patient were *CDKN2A*, *AR*, *CDKN2B*, *ESR1*, *KRAS*, *CDK4*, *PIK3CA*, *AKT2*, *TP53*, and *CCND1*. Notably, copy number events comprised 78% of the 58 concordant events, corresponding to 38 of the 50 patients. Concordant events exhibited a Spearman correlation of 0.72 between copy number and expression, as shown in Supplementary Fig. 3. The remaining 22% of events were all missense mutations (*N* = 12), except for one fusion and one splice site variant (Fig. 2b and Supplementary Table 3).

The number of altered genes by tumor type for the patients exhibiting concordant DNA and RNA alterations is shown in Supplementary Fig. 1. Concordance did vary by tumor type. Although colorectal cancer was the most prevalent tumor type (21% of the 253 patients), only one patient with colorectal cancer exhibited concordance. Gastrointestinal cancer and breast cancer had higher levels of concordance (30% and 25%, respectively) relative to the cohort average of 20%, and head and neck cancer and sarcoma had intermediate concordance rates (7% and 13%, respectively). However, concordance status also varied by tumor purity (Supplementary Fig. 2), and samples from patients with concordant events had higher tumor purity (*p* = 0.001, Mann-Whitney U test).

### Significant associations between alterations identified by DNA and RNA profiling

**Fig. 3:**
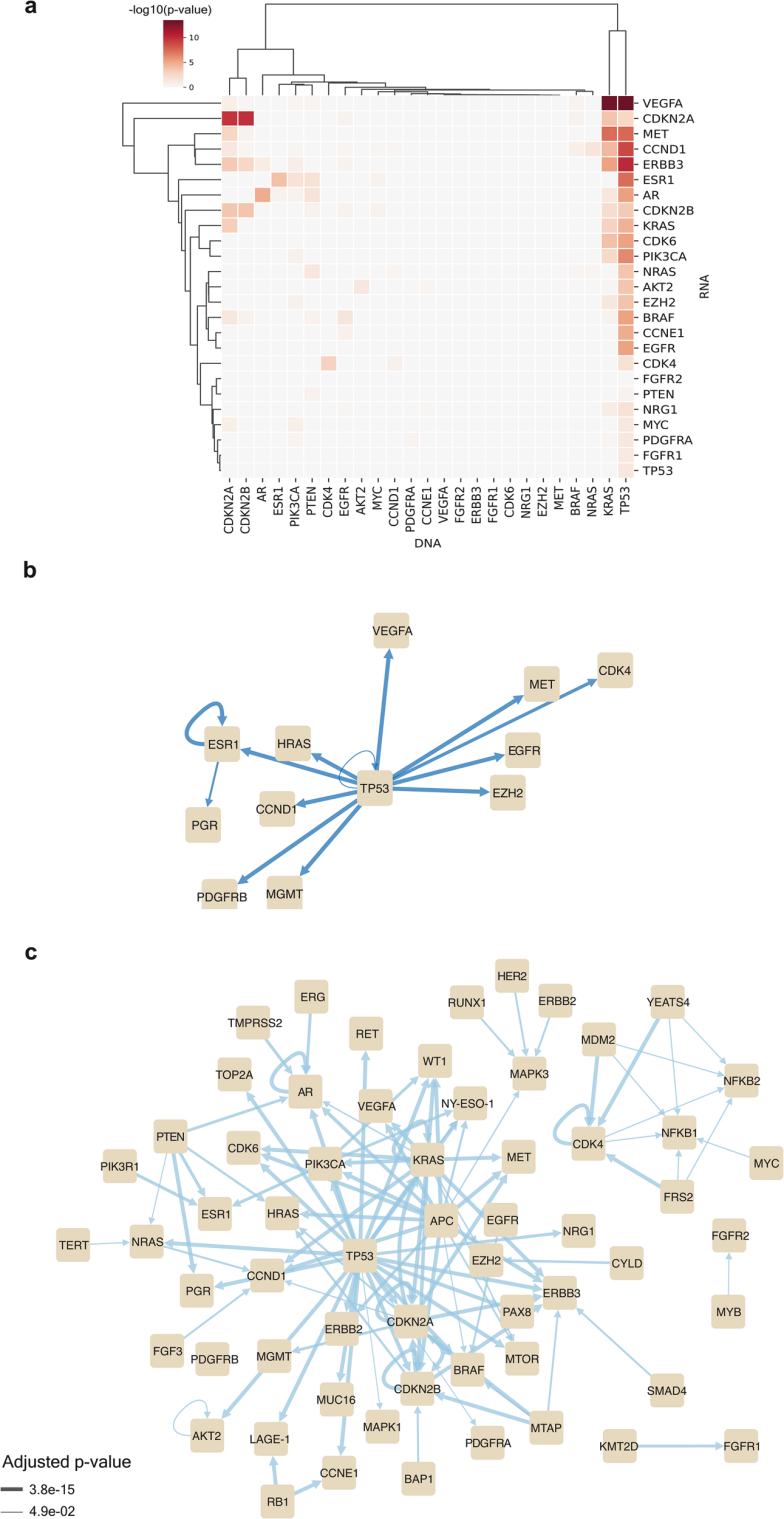
Association between genes reported as altered by DNA profiling and those reported as altered by RNA profiling. **a** Gene pairs with significant concordance are shown; darker shading indicates a more significant association [-log_10_(adjusted *p*-value)] for the indicated gene pair (all gene pairs are shown in Supplemental Fig. 1). **b** Network of gene pairs with significant associations comprised of transcription factors and their targets. Genes with genomic alterations are the “edge sources”, while genes with altered expression serve as the “edge targets”. “Edge weight” denotes significance of association. Notably, “edge sources” refer to the source node of an edge, while “edge targets” refer to the end of an edge. “Edge weight” refers here to the line thickness. **c** Network of all other significantly associated gene pairs (i.e., not transcription factors and their targets)

The association between genomic alterations and altered expression was determined for the 12,584 pairs comprised of each gene reported by DNA profiling and each gene reported by RNA profiling. Overall, the DNA and RNA profiling results were significantly associated in 123 gene pairs (adjusted *p*-value < 0.05), as shown in Fig. 3a and Supplementary Fig. 4. Eight of the 123 pairs meeting statistical significance were concordant pairs involving the following genes: *AR*, *AKT2*, *CDK4*, *CDKN2A*, *CDKN2B*, *ESR1*, *KRAS*, and *TP53*; these pairs were observed in 41 patients.

Pathway annotation of the genes involved in the 123 significant DNA-RNA pairs demonstrated strong associations with the PI3K/AKT signaling pathway (adjusted *p*-value = 5.87e−30), as shown in Table 2. Additionally, 13 pairs were comprised of a transcription factor with a genomic alteration and one of its targets with altered expression (Fig. 3b). Interestingly, the 3 most significant DNA-RNA gene pairs were *VEGFA* overexpression with *KRAS*, *TP53*, or *APC* molecular alterations.

**Table 2:** Top 10 Reactome pathways associated with genes from significant DNA-RNA pairs

Gene Set Name	Adjusted *p*-value
REACTOME_PI3K_AKT_SIGNALING_IN_CANCER	5.87E−30
REACTOME_NEGATIVE_REGULATION_OF_THE_PI3K_AKT_NETWORK	1.1E−29
REACTOME_CONSTITUTIVE_SIGNALING_BY_ABERRANT_PI3K_IN_CANCER	2.65E−23
REACTOME_INSULIN_RECEPTOR_SIGNALLING_CASCADE	1.35E−21
REACTOME_SIGNALING_BY_FGFR1	1.72E−19
REACTOME_ESR_MEDIATED_SIGNALING	1.72E−19
REACTOME_SIGNALING_BY_INSULIN_RECEPTOR	1.81E−19
REACTOME_SIGNALING_BY_FGFR	4.5E−19
REACTOME_SIGNALING_BY_ERBB2_IN_CANCER	3.89E−18
REACTOME_SIGNALING_BY_FGFR2	4.95E−18

### Tumor transcriptional burden and overall survival

**Fig. 4:**
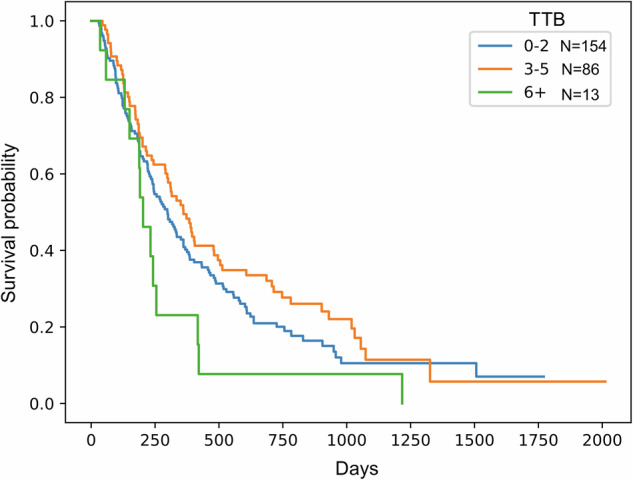
Kaplan-Meier curves of patient overall survival stratified by tumor transcriptional burden (TTB)

The impact of TTB on OS is shown in Fig. 4. Patients with higher TTB had poorer outcomes; the median OS durations for patients with 0 to 2, 3 to 5, and ≥6 genes with altered expression were 9.8, 11.9, and 6.7 months, respectively (*p* = 0.03, log-rank test).

**Fig. 5:**
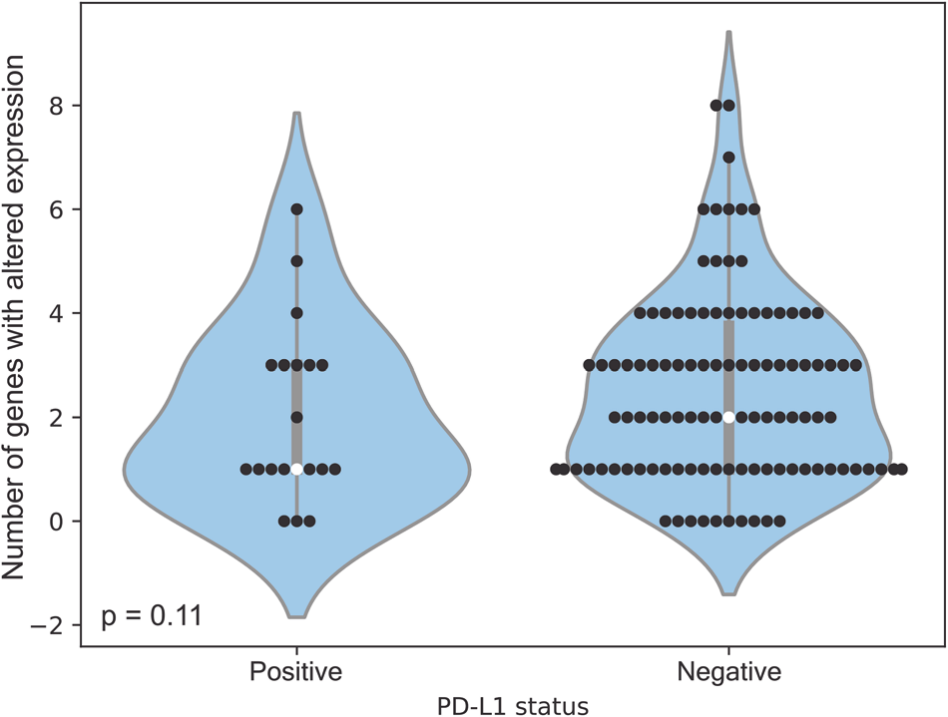
Distribution of genes with altered expression by PD-L1 status. The medians are indicated by the white points

Given the importance of PD-L1 in the efficacy of immunotherapy, TTB was also considered in relation to the PD-L1 status of patients (Fig. 5). Patients whose tumors were PD-L1 negative tended to have a greater number of genes with altered expression than those whose tumors were PD-L1 positive (*p* = 0.11, Mann-Whitney U test).

## Discussion

This study represents, to our knowledge, the first comprehensive evaluation of RNA expression profiling in conjunction with DNA-based molecular analysis within the setting of a prospective precision oncology clinical trial across tumor types. By retrospectively analyzing data from the prospective IMPACT2 study, we uniquely explored the concordance between genomic and transcriptomic alterations as well as their association with OS. First, we found significant concordance between DNA and RNA alterations in a subset of genes, namely *AR*, *AKT2*, *CDK4*, *CDKN2A*, *CDKN2B*, *ESR1*, *KRAS*, and *TP53*. Second, we observed a significant association between *TP53* alterations and *VEGFA* overexpression, which may in part explain tumor response to bevacizumab in *TP53*-mutant patients. Third, we discovered that TTB ≥ 6 is associated with shorter OS. The integration of DNA and RNA data from patients treated under real-world conditions allowed for a nuanced examination of how these molecular layers interact and influence clinical outcomes, an approach not previously reported in the context of a precision oncology trial.

While DNA-based molecular profiling has transformed the treatment paradigm for advanced cancer by guiding the selection of FDA-approved or investigational targeted therapies, the dynamic and complex nature of tumor biology is not fully captured by genomic alterations alone.^9^ RNA profiling can provide complementary information that reflects active oncogenic signaling, microenvironmental interactions, and downstream transcriptional effects, which are not necessarily reflected in static DNA alterations. Our findings suggest that RNA profiling can provide actionable insights for the clinic beyond those offered by DNA profiling, particularly in tumors lacking canonical actionable mutations.

First, in this analysis of patients enrolled in the prospective IMPACT2 trial, we identified significant concordance between DNA and RNA alterations in a subset of genes, namely *AR*, *AKT2*, *CDK4*, *CDKN2A*, *CDKN2B*, *ESR1*, *KRAS*, and *TP53*. These genes are recognized drivers in multiple tumor types, and the predominance of copy number events among these concordant DNA-RNA alterations supports prior observations that gene amplifications frequently translate into RNA overexpression.^7^

In contrast, there were 12,526 observed discordant events (i.e., those involving DNA and RNA alterations in different genes), which were tested for significance of association. Of 12,526 observed discordant events, 115 events displayed significant association. Since according to protocol design, RNA profiling was investigational and it was not used for treatment selection, the gene alterations from discordant events were not considered actionable.

The fact that concordance status varied by tumor purity suggests that there may have been additional concordant events present that were not observed in tumors with low purity. Therefore, while there appear to be differences between tumor types with respect to the concordance of DNA and RNA alterations, additional parameters such as tumor purity contribute to whether concordant events are observed. The observation that not all patients exhibit concordance between DNA and RNA profiling also aligns with previous studies demonstrating that many DNA alterations are not reflected in RNA profiling due to regulatory or post-transcriptional factors^10–12^ and further underscores the added value of directly measuring transcriptional activity rather than inferring it from the genome.

Second, an intriguing finding from our study was the interaction between *TP53* and *VEGFA*, one of the most significantly associated DNA-RNA alteration pairs. *VEGFA* is a known transcriptional target of *TP53*, and its overexpression when *TP53* is inactivated may explain in part the observed benefit of anti-angiogenic therapies such as bevacizumab in patients with *TP53*-mutant tumors.^13,14^ This reinforces the potential of RNA profiling to uncover clinically relevant gene-gene interactions that are not readily evident from genomic data alone and that can inform future biomarker-driven therapeutic strategies.

Third, we found that TTB was inversely associated with OS. Patients with ≥6 altered genes had significantly shorter OS than those with fewer altered genes, and patients with higher TTB tended to be PD-L1 negative. These observations suggest that global transcriptomic dysregulation may reflect more aggressive tumor biology or resistance to therapy, which is consistent with recent reports linking transcriptional signatures to clinical outcomes across cancer types.^15,16^ The optimal cut-off for TTB may not be reflected in this pilot study and should be determined in a large number of patients with complete DNA and RNA profiling. These results provide support for TTB as a candidate prognostic biomarker that warrants further investigation.

Finally, although the association of PD-L1–negative tumors with high TTB (Fig. 5) did not reach statistical significance (*p* = 0.11), this may suggest that tumors with high TTB represent a more aggressive, immune-excluded phenotype, which should be tested in a larger number of patients. This is consistent with the reported tumor biology from pan-cancer analyses wherein oncogenic transcriptional programs actively drive immune exclusion.^17^ Notably, the association between PD-L1 and TTB warrants validation in larger cohorts.

Taken together, our results suggest that RNA profiling provides clinical utility and may be of particular benefit to patients without actionable genomic alterations. While RNA-based tests have yet to gain regulatory approval for treatment selection, several studies have shown that transcriptomic classifiers can identify patient subgroups likely to benefit from immunotherapy or targeted agents.^18,19^ Moreover, RNA-based biomarkers have been incorporated into molecular diagnostics in other settings, including the Oncotype DX assay for early-stage breast cancer.^20^ Such developments support the broader feasibility of RNA profiling in routine oncology care.

We have previously reported on the WINTHER trial (NCT01856296) that used DNA and RNA profiling to select treatment for patients with advanced cancer across tumor types and showed that transcriptomics increased the fraction of patients who could receive matched therapy when there were no actionable DNA alterations or when drugs selected on the basis of DNA alterations were unavailable.^21^ Compared to the IMPACT2 trial, the WINTHER trial used a more limited gene panel (owing to the timing of the study) and the concordance between DNA and RNA profiling was not analyzed. Still, this prior work suggested that both genomic and transcriptomic profiling can be useful in guiding treatment recommendations and, along with future trials, can help establish the use of transcriptomic profiling in the clinic.

In the WINTHER trial, RNA profiling was performed using Agilent microarray technology to compare RNA expression in tumor samples to that in matched normal tissue for treatment selection. The differences between the RNA profiling performed in the WINTHER trial and IMPACT2 study are summarized in Supplementary Table 4. In the WINTHER trial, RNA profiling improved the selection of matched targeted therapy from 23% achieved with DNA profiling alone to 35% with the addition of RNA profiling results. However, biopsies of paired tumor and normal tissue from the same organ were required, which represented a major challenge, as normal tissue is not always available in patients with metastatic cancer owing to the origin of the cancer (e.g., pancreas) or to the increased risk associated with dual biopsies (e.g., lung cancer).

In addition, therapeutic matching was performed in the WINTHER trial through a decision-making algorithm that used differential expression between tumor and normal tissue to create an expression profile that was then compared against a comprehensive proprietary drug knowledge database to generate a ranked list of potential therapies for each patient.

In IMPACT2, RNA profiling was performed by Tempus (xT assay), which uses next-generation sequencing–based whole transcriptome RNA sequencing from tumor tissue without matched normal tissue comparison. While this precluded direct assessment of tumor-specific differential expression, as in the WINTHER trial, it has the advantage of not requiring a biopsy of normal tissue, and standard FFPE specimens can be used for testing.

The current analysis demonstrates concordance between DNA alterations and RNA expression, suggesting that tumor RNA profiling without matched normal tissue can provide valuable complementary information that may be considered for investigation in future clinical trials (Supplementary Table 4).

The limitations of our study include the retrospective nature of the analysis embedded within a prospective clinical trial. The cohort was also heterogeneous with respect to tumor type, molecular profiles, and prior treatment, which may have confounded associations between TTB and survival. With respect to the interplay between tumor type and concordance, only one concordant event was observed in *ERBB2* in a patient with endometrial cancer. This cannot be explained by the distribution of tumor types in patients with both DNA and RNA profiling, since *ERBB2* amplification with corresponding overexpression is expected in endometrial and other gynecologic cancers, which comprised 8% of patients with concordant events. Of these four patients, only one exhibited concordance in *ERBB2*, while two patients exhibited concordance in *TP53* and the remaining exhibited concordance in *CDKN2A*, *CDKN2B*, and *EGFR*. However, because our cohort is comprised of heavily pretreated patients with advanced disease, additional alterations can complicate the detection of concordant signals, which may be the case for *ERBB2*. With respect to hormone-specific concordance findings, we observed that among the nine patients with *AR* concordance, all had prostate cancer. Similarly, *ESR1* concordance was observed in five patients with breast cancer, consistent with the known biology of hormone receptor-driven tumors. Both *AR* and *ESR1* are involved in the PI3K/AKT signaling pathway, which was the most significantly enriched pathway in our analysis (adjusted *p*-value = 5.87e−30) as noted in the Results section. No correlation between *AR* or *ESR1* and *EGFR* was noted. However, both pathways ultimately converge on PI3K/AKT signaling, suggesting complex interconnected networks that may be better captured through integrated multi-omic profiling. Future prospective studies that integrate RNA profiling into treatment decision algorithms are warranted to validate the findings of this study and support the use of RNA profiling as a complementary approach in the clinical setting.

In summary, RNA profiling captures biological complexity that DNA profiling on its own may not and, accordingly, holds promise for guiding clinical decision-making. Our results demonstrate that TTB is associated with OS. Further studies should validate our findings and specifically assess the utility of RNA profiling in complementing DNA-based approaches for treatment selection and outcome prediction. With the use of artificial intelligence and machine learning techniques, RNA profiling can be integrated into DNA and other biomarker analyses to optimize treatment selection.^22^ As transcriptomics become more accessible and standardized, their integration into precision oncology frameworks may help refine therapeutic strategies and improve patient outcomes.

## Methods

### Molecular testing

All IMPACT2 study participants had provided written informed consent before enrollment stating that they were aware of the investigational nature of the study. The applicable study protocol was approved by the Institutional Review Board at The University of Texas MD Anderson Cancer Center on May 13, 2014 (Registration Number: IRB 1 IRB00000121) and was registered on ClinicalTrials.gov (NCT02152254).

Of the 829 patients studied in IMPACT2, 438 consecutive patients were enrolled in Part B of the trial from April 2019 until October 2023 and were scheduled to undergo a tumor biopsy and genomic profiling of the tumor tissue by Tempus using the Tempus xT assay. DNA profiling and PD-L1 testing were prioritized, and RNA profiling was performed for the 253 patients with adequate remaining tumor tissue. The assay and downstream bioinformatic analysis were performed as previously reported^23^ (Supplemental File). The analyses performed were based on the DNA and RNA profiling reports provided by Tempus and were not restricted to only pathogenic variants.

### Data handling and analysis set-up

Processed DNA and RNA profiling data were analyzed in Python using the jupyter,^24^ numpy,^25^ pandas,^26^ scipy,^27^ lifelines,^28^ matplotlib,^29^ seaborn,^30^ and networkx^31^ packages.

### Concordance analysis of DNA and RNA profiling

The pairing of each gene reported by DNA profiling and each gene reported by RNA profiling was evaluated for significance of association by applying Fisher’s exact test^32^ to the following contingency table: (1) Patients *without* genomic alteration in the selected gene from DNA profiling and *without* altered expression in the selected gene from RNA profiling; (2) Patients *with* genomic alteration in the selected gene from DNA profiling but *without* altered expression in the selected gene from RNA profiling; (3) Patients *without* genomic alteration in the selected gene from DNA profiling but *with* altered expression in the selected gene from RNA profiling; and (4) Patients *with* genomic alteration in the selected gene from DNA profiling and *with* altered expression in the selected gene from RNA profiling. Benjamini-Hochberg correction^33^ was subsequently performed across all pairs.

### Pathway annotation

Annotation of the genes in pairs that met statistical significance was performed using gene set enrichment analysis with the Canonical Pathways: Reactome collection.^34^ Gene sets with at least 10 genes and fewer than 250 genes were included in the testing, and those with an overlap of at least 10 genes with the query genes and with an adjusted *p*-value < 0.05 were reported.

### Transcription factor annotation

The Transcriptional Regulatory Relationships Unraveled by Sentence-based Text mining (TRRUST), version 2, database was used to annotate genes of interest as transcription factors or their targets.^35^

### Statistical analysis

Kaplan-Meier methodology^36^ was used to analyze OS in the 217 treated patients with RNA profiling results. Patients were stratified by the number of genes with altered expression, as follows: 0 to 2 genes, 3 to 5 genes, and ≥6 genes. OS among the groups was compared using the log-rank test.^37^

The cut-off for statistical significance used throughout was *p* < 0.05. PD-L1 status and number of genes with altered expression were evaluated using the Mann-Whitney U test.^38^

## Supplementary information

Supplementary Materials

## Data Availability

The data supporting the findings of this study are available within the Main Text and Supplementary Materials. All requests for further data sharing will be reviewed by MD Anderson and the study sponsors to determine whether the request is subject to any intellectual property or confidentiality obligations. For further questions, please contact the corresponding author, Dr. Apostolia Maria Tsimberidou (atsimber@mdanderson.org).
