# Supplementary material for: Concordance analysis of DNA and RNA profiling: The MD Anderson IMPACT2 study in precision oncology
Source: Signal Transduct Target Ther. 2026 Feb 24;11:68. doi: 10.1038/s41392-026-02580-0 (PMC12929784; doi:10.1038/s41392-026-02580-0)
Supplement: Supplementary file 1 — Supplementary Materials [file 41392_2026_2580_MOESM1_ESM.docx]

Supplementary Materials for

Concordance analysis of DNA and RNA profiling: Results of the MD Anderson

IMPACT2 study in precision oncology

Stephanie T. Schmidt, Mehmet A. Baysal, Siqing Fu, David S. Hong, Sarina A. Piha-Paul,

Aung Naing, Jordi Rodon Ahnert, Timothy A. Yap, Ecaterina Elena Dumbrava, Jennifer Beck, Funda Meric-Bernstam, Apostolia Maria Tsimberidou

Correspondence to: atsimber@mdanderson.org

**This PDF file includes:**

Patients and Methods

Figures. S1-S4

Tables S1-S4

# Patients and Methods

## Eligibility criteria

Patients had to have measurable disease, biopsy-accessible tumor, and normal organ and marrow function (absolute neutrophil count ≥ 1,000/µl; platelets ≥ 100,000/µl), adequate hepatic function (bilirubin ≤ 1.5 x upper limit of normal [ULN]; alanine transaminase ≤ 2.5 x ULN), and serum creatinine clearance ≥ 50 ml/min (Cockcroft-Gault formula). Additional eligibility criteria included no brain metastasis or treated, stable, and asymptomatic brain metastasis for ≥ 4 weeks (off steroids ≥ 2 weeks). If patients had previous malignancies, they had to be disease-free for ≥ 3 years.

Patients are excluded from this study if they have prior chemotherapy, surgery, or radiotherapy within 3 weeks of initiating study treatment. Additional exclusion criteria include severe cardiac conditions, peripheral neuropathy ≥ grade 2, and concurrent severe and/or uncontrolled medical disease that could compromise participation in the study. Patients are also excluded if they have refractory nausea, vomiting, and/or chronic gastrointestinal diseases or have undergone a significant bowel resection that would preclude adequate absorption (for oral therapy only).

Genomic and Transcriptomic Analysis

Genomic testing was performed at Tempus (Chicago, IL) using the Tempus xT Oncology Assay, which included a 596-gene targeted sequencing panel from April 2019 to October 2019 and a 648gene targeted sequencing panel starting in November 2019. Formalin-fixed, paraffin-embedded (FFPE) tumor samples and matched normal blood or saliva samples were analyzed to detect somatic single-nucleotide variants (SNVs), insertion-deletions (indels), copy number variants (CNVs), gene rearrangements, and microsatellite instability (MSI).

Library preparation was performed using the KAPA HyperPrep Kit, followed by target capture with custom-designed Roche probes. Sequencing reads were aligned to the GRCh37 human reference genome using NovoAlign and the Burrows–Wheeler Aligner (BWA). Variant calling was conducted, and the resulting variant call format (VCF) files were annotated using the SnpEff R package. Variants were classified as either somatic or germline. CNVs were identified using a proprietary tumor/normal-matched analysis implemented via the R package CNATools.

Tumor mutational burden (TMB) was calculated and reported as mutations per megabase (Mut/Mb). MSI status and PD-L1 protein expression were also assessed; PD-L1 expression was quantified via immunohistochemistry and reported as Tumor Proportion Score (TPS). TMB values were considered actionable if they met or exceeded thresholds defined by FDA approvals, clinical guidelines, or active clinical trials at the time of interpretation.

In addition to DNA-based testing, whole-transcriptome RNA sequencing was performed as part of the Tempus xT assay for exploratory analyses, including expanded detection of gene rearrangements, immune cell infiltration, and expression profiling of immune- and targeted– therapy–related genes. RNA library preparation was also performed using the KAPA HyperPrep

Kit, followed by capture using IDT xGen Lockdown® Probes, Exome Research Panel v1.0. Sequencing reads were aligned to the GRCh38 reference genome using STAR. Transcript abundances were quantified as transcripts per million (TPM) using Kallisto, and gene-level expression counts were generated using FeatureCounts.


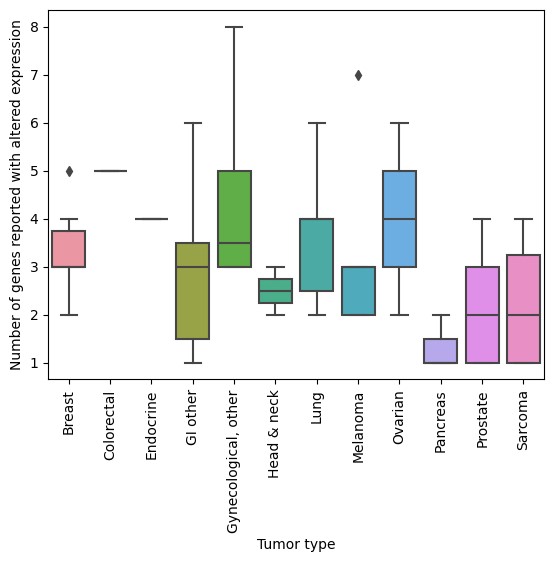


**Figure. S1.** Number of altered genes by tumor type in patients with concordant DNA and RNA alterations. Each box denotes the interquartile range and includes the median line, while the whiskers extend to the minimum and maximum values 1.5 times the interquartile range. Data points beyond the whiskers are shown by individual points.


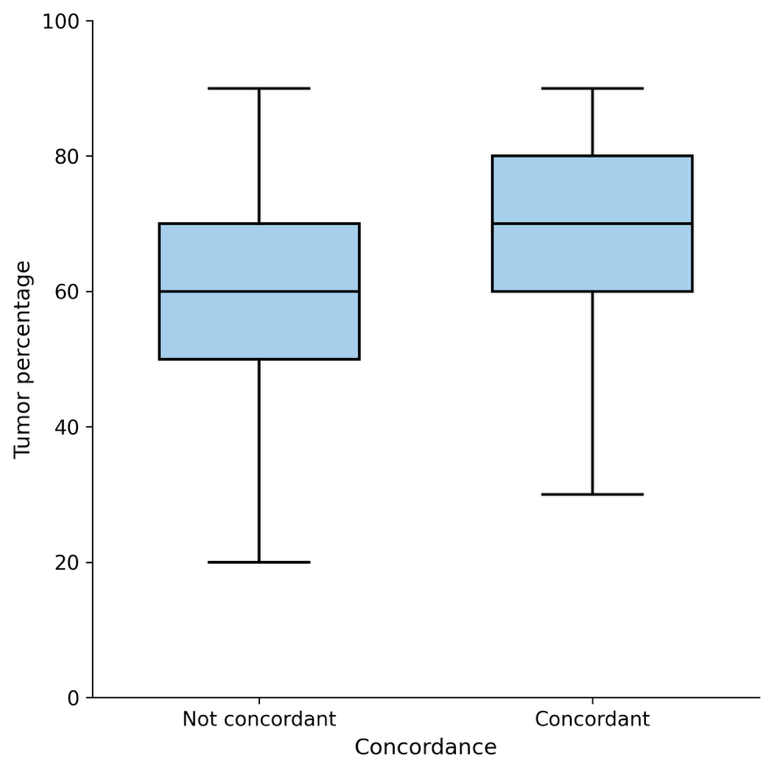


**Figure. S2.** Tumor percentage by concordance status. Each box denotes the interquartile range and includes the median line, while the whiskers extend to the minimum and maximum values 1.5 times the interquartile range.


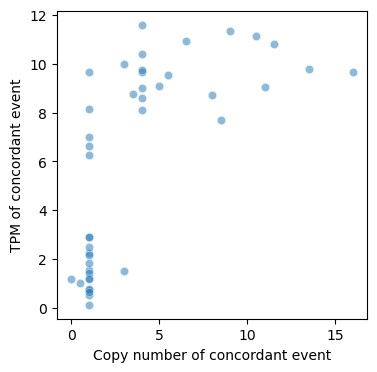


**Figure. S3.** RNA expression (in transcripts per million, TPM) versus copy number for concordant copy number events.


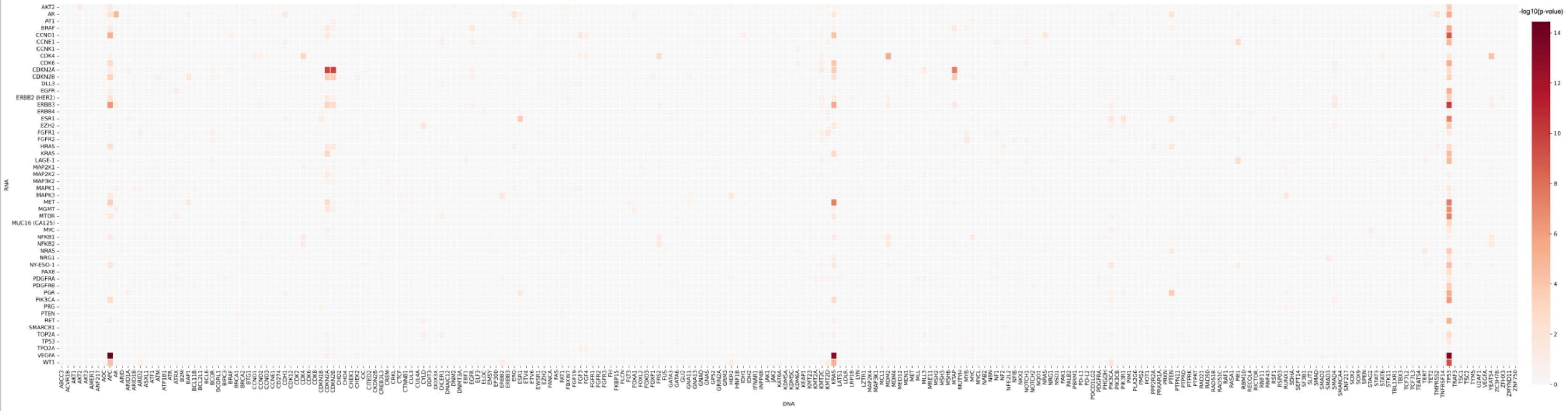

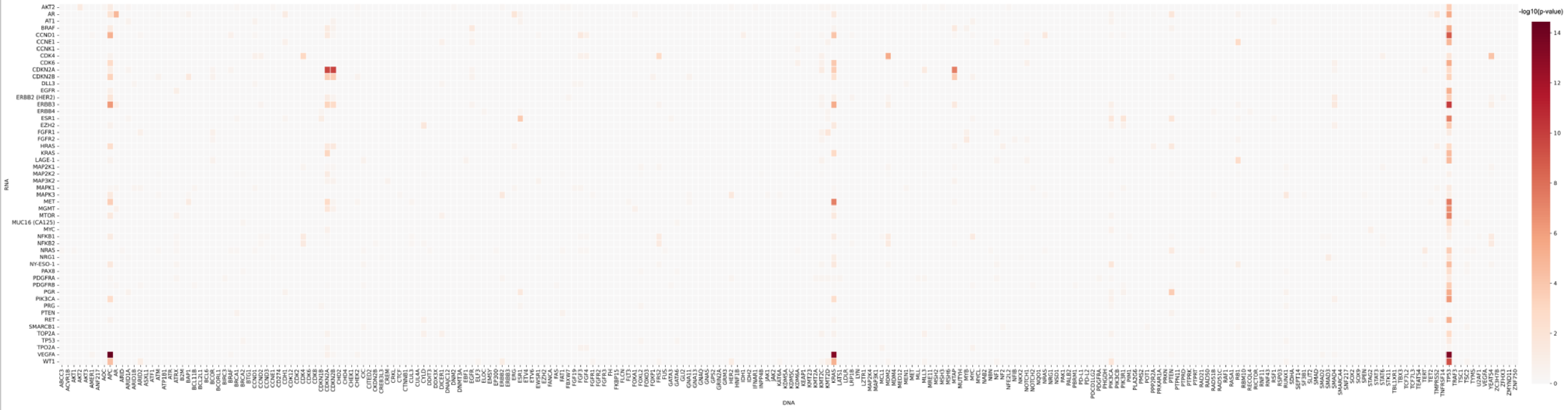


**Figure. S4.** Full heatmap of associations between genetic and expression alterations. Genes with DNA alterations are shown on the x-axis, and those with RNA alterations are shown on the y-axis. (Page 1 of 2)

**Figure. S4.** Full heatmap of associations between genetic and expression alterations. Genes with DNA alterations are shown on the x-axis, and those with RNA alterations are shown on the y-axis. (Page 2 of 2)


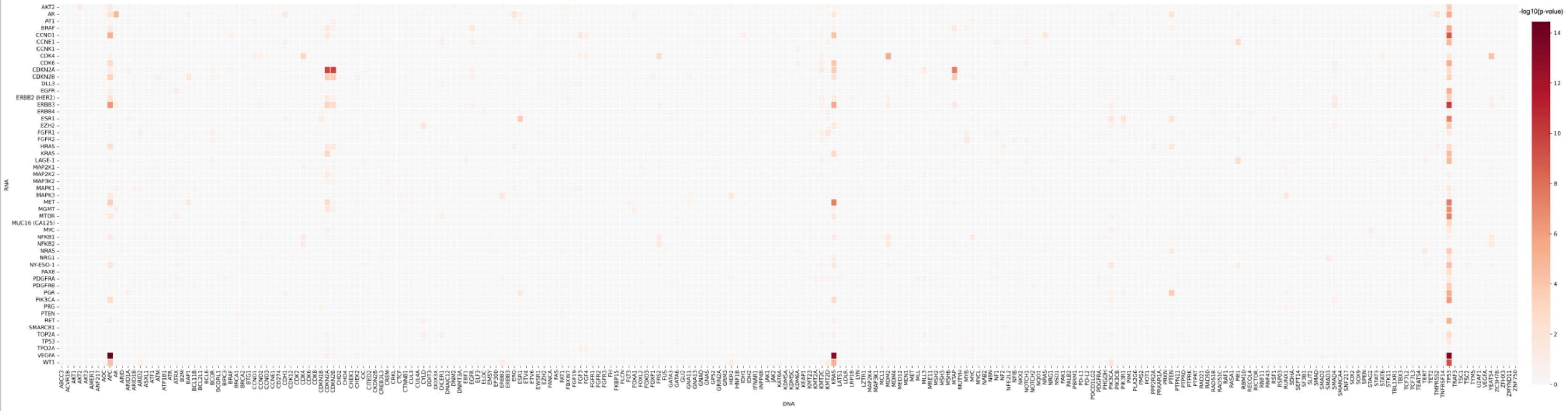

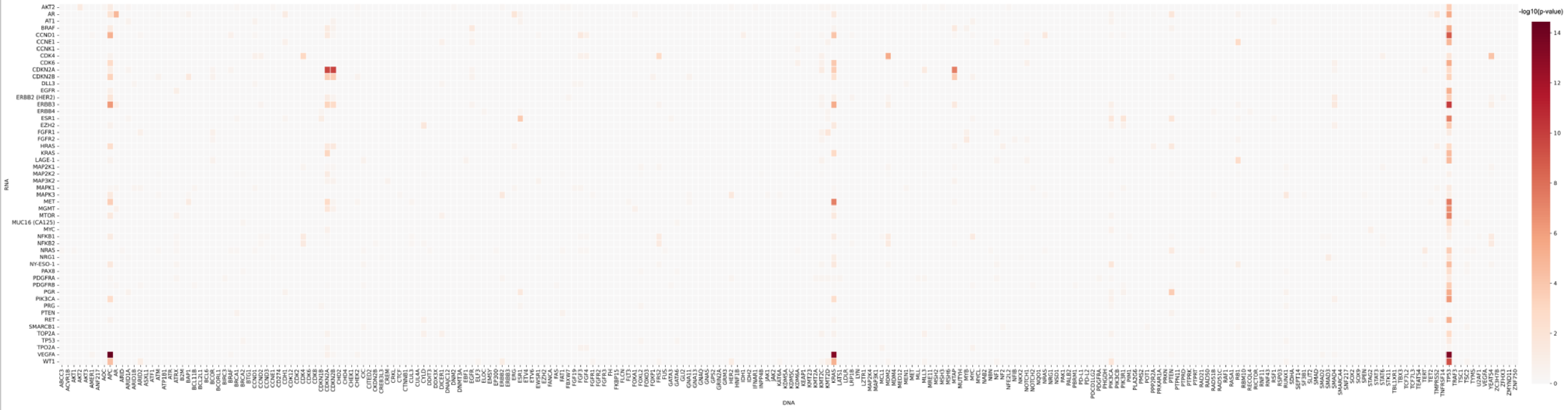


**Table S1.** DNA and RNA profiling (N = 253), altered expression (N = 237), and concordant events (N = 50) by tumor type.

|  | **Patients with**  **DNA and RNA**  **profiling (N=253)** | | **Patients with altered expression (N=237)** | | **Patients with concordant events (N=50)** | |
| --- | --- | --- | --- | --- | --- | --- |
| **Tumor Type** | Number | Percent | Number | Percent | Number | Percent |
| Brain | 2 | 1 | 2 | 1 | 0 | 0 |
| Breast | 24 | 9 | 24 | 10 | 6 | 12 |
| Colorectal | 53 | 21 | 48 | 20 | 1 | 2 |
| Endocrine | 5 | 2 | 3 | 1 | 1 | 2 |
| Genitourinary (non-Prostate) | 0 | 0 | 0 | 0 | 0 | 0 |
| Gastrointestinal (non-Colorectal) | 23 | 9 | 18 | 8 | 7 | 14 |
| Gynecologic (non-Ovarian) | 8 | 3 | 7 | 3 | 4 | 8 |
| Head & Neck | 30 | 12 | 26 | 11 | 2 | 4 |
| Lung | 13 | 5 | 26 | 11 | 6 | 12 |
| Melanoma | 15 | 6 | 12 | 5 | 5 | 10 |
| Mesothelioma | 4 | 2 | 3 | 1 | 0 | 0 |
| Ovarian | 9 | 4 | 8 | 3 | 2 | 4 |
| Pancreas | 17 | 7 | 15 | 6 | 3 | 6 |
| Prostate | 18 | 7 | 18 | 8 | 9 | 18 |
| Sarcoma | 30 | 12 | 25 | 11 | 4 | 8 |
| Skin (non-Melanoma) | 1 | 0.4 | 1 | 0.4 | 0 | 0 |
| Cancer of Unknown Primary | 1 | 0.4 | 1 | 0.4 | 0 | 0 |

**Table S2.** Distribution of expression alteration by count and directionality.

| **Number of overexpressed genes** | **Number of underexpressed genes** | **Number of patients with expression pattern** |
| --- | --- | --- |
| 0 | 0 | 28 |
| 0 | 1 | 12 |
| 0 | 2 | 2 |
| 1 | 0 | 38 |
| 1 | 1 | 8 |
| 1 | 2 | 4 |
| 2 | 0 | 52 |
| 2 | 1 | 8 |
| 2 | 2 | 1 |
| 2 | 6 | 1 |
| 3 | 0 | 51 |
| 3 | 1 | 5 |
| 3 | 2 | 2 |
| 3 | 3 | 1 |
| 4 | 0 | 20 |
| 4 | 1 | 4 |
| 5 | 0 | 10 |
| 5 | 1 | 2 |
| 5 | 2 | 2 |
| 6 | 0 | 2 |

**Table S3**. Summary of concordant events by patient with associated metadata.

| **Patient**  **Number** | **Tumor type** | **Biopsy Location** | **Tumor percent** | **Concordant genes** | **DNA**  **alterations** | **RNA**  **expression** |
| --- | --- | --- | --- | --- | --- | --- |
| 1 | Prostate | Pelvic Lymph Node | 60 | AR | MISSENSE | OVER |
| 2 | Sarcoma | Retroperitoneal Lymph Node | 70 | AKT2 | GAIN | OVER |
| 3 | Prostate | Retroperitoneum | 70 | AR | GAIN | OVER |
| 4 | Gynecological | Lymph Node | 90 | EGFR,  CDKN2A,  CDKN2B | GAIN, LOSS, LOSS | OVER,  UNDER,  UNDER |
| 5 | Prostate | Retroperitoneal Lymph Node | 80 | AR | MISSENSE | OVER |
| 6 | Prostate | Retroperitoneal Lymph Node | 90 | AR | GAIN | OVER |
| 7 | Breast | Liver | 50 | ESR1 | MISSENSE | OVER |
| 8 | Head & neck | Neck Lymph Node | 70 | PIK3CA | GAIN | OVER |
| 9 | Gynecological | Abdominal Wall Soft Tissue | 40 | ERBB2 | GAIN | OVER |
| 10 | Breast | Axilla Lymph Node | 50 | FGFR1 | GAIN | OVER |
| 11 | Breast | Liver | 60 | ESR1 | MISSENSE | OVER |
| 12 | Breast | Liver | 60 | ESR1 | MISSENSE | OVER |
| 13 | Breast | Liver | 70 | ESR1 | MISSENSE | OVER |
| 14 | Head & neck | Neck Soft Tissue | 70 | PIK3CA | MISSENSE | OVER |
| 15 | Lung | Lung | 80 | CDKN2A,  CDKN2B, BRAF | LOSS, LOSS, GAIN | UNDER,  UNDER,  OVER |
| 16 | Prostate | Liver | 60 | AR | LOSS | OVER |
| 17 | Endocrine | Abdomen Soft Tissue | 80 | CDK4 | GAIN | OVER |
| 18 | Pancreas | Lung | 60 | KRAS | MISSENSE | OVER |
| 19 | Sarcoma | Chest Wall Soft Tissue | 70 | CDKN2B | LOSS | UNDER |
| 20 | Lung | Liver | 80 | CDKN2A | LOSS | UNDER |
| 21 | Lung | Neck Soft Tissue | 60 | CCNE1 | GAIN | OVER |
| 22 | GI | Liver | 40 | KRAS | GAIN | OVER |
| 23 | GI | Abdomen Soft Tissue | 50 | KRAS | MISSENSE | OVER |
| 24 | Prostate | Retroperitoneal Lymph Node | 90 | AR | GAIN | OVER |
| 25 | Sarcoma | Liver | 70 | CDK4 | GAIN | OVER |
| 26 | GI | Liver | 80 | FGFR2 | FUSION | OVER |
| 27 | Lung | Liver | 80 | CDKN2A | LOSS | UNDER |
| 28 | Ovarian | Inguinal Lymph Node | 80 | KRAS | GAIN | OVER |
| 29 | Gynecological | Liver | 80 | TP53 | MISSENSE | OVER |
| 30 | Lung | Scapula Soft Tissue | 80 | CDK6 | GAIN | OVER |
| 31 | Prostate | Supraclavicular Lymph Node | 80 | AR | GAIN | OVER |
| 32 | Prostate | Retroperitoneal Lymph Node | 70 | AR | GAIN | OVER |
| 33 | Prostate | Lung | 60 | AR,AR | GAIN, MISSENSE | OVER, OVER |
| 34 | GI | Supraclavicular Lymph Node | 60 | MET | GAIN | OVER |
| 35 | Lung | Bronchus | 70 | MYC | GAIN | OVER |
| 36 | Gynecological | Retroperitoneal Lymph Node | 80 | TP53 | SPLICE  SITE  VARIANT | OVER |
| 37 | Melanoma | Lung | 80 | CDKN2A, CDKN2B | LOSS, LOSS | UNDER, UNDER |
| 38 | Pancreas | Liver | 60 | CDKN2A | LOSS | UNDER |
| 39 | Pancreas | Lung | 50 | CDKN2A | LOSS | UNDER |
| 40 | GI | Liver | 60 | CCND1 | GAIN | OVER |
| 41 | Melanoma | Abdomen Soft Tissue | 70 | CDKN2A | LOSS | UNDER |
| 42 | Melanoma | Pelvic Lymph Node | 30 | PDGFRA | GAIN | OVER |
| 43 | GI | Retroperitoneal Lymph Node | 70 | CCND1, NRAS | GAIN, GAIN | OVER, OVER |
| 44 | Melanoma | Inguinal Lymph Node | 70 | CDKN2A | LOSS | UNDER |
| 45 | Breast | Liver | 70 | ESR1 | MISSENSE | OVER |
| 46 | Colorectal | Pelvis | 70 | AKT2 | GAIN | OVER |
| 47 | Sarcoma | Abdomen | 90 | CDK4 | GAIN | OVER |
| 48 | Melanoma | Abdominal Wall Soft Tissue | 70 | CDKN2A, CDKN2B | LOSS, LOSS | UNDER, UNDER |
| 49 | Ovarian | Liver | 70 | PTEN | LOSS | UNDER |
| 50 | GI | Liver | 80 | CDKN2A | LOSS | UNDER |

**Table S4.** Differences in RNA profiling and analysis between the WINTHER and IMPACT2 studies.

| **Process/Impact** | **WINTHER** | **IMPACT2** |
| --- | --- | --- |
| Samples required | Fresh tumor biopsy, matched normal tissue biopsy. | Tumor tissue (FFPE); no normal tissue biopsy used. |
| Methodology | Agilent oligonucleotide microarrays. | Whole transcriptome RNA sequencing through Tempus xT assay. |
| Normal tissue requirement | Yes, direct tumor-matched normal tissue for comparison with tumor tissue. | No, tumor only (no matched normal tissue was required for comparison with tumor). |
| Application | Used to select treatment in cases where DNA profiling was not actionable. | Exploratory; not used for treatment selection. |
| Interpretation | Algorithm linking differential expression to drug knowledge database, required for analysis. | Commercially available RNA profiling; does not require comparison with drug knowledge database. |
| Analysis | RNA analysis results were used for treatment selection (added to endpoint of the study). | *Ad hoc* correlation with DNA analysis, not an endpoint of the study. |
| Impact | First prospective trial using transcriptomics for treatment selection. | Exploratory analysis. |
